# Selective Transurethral Resection of the Prostate Combined with Transurethral Incision of the Bladder Neck for Bladder Outlet Obstruction in Patients with Small Volume Benign Prostate Hyperplasia (BPH): A Prospective Randomized Study

**DOI:** 10.1371/journal.pone.0063227

**Published:** 2013-05-14

**Authors:** Xin Li, Jin-hong Pan, Qi-gui Liu, Peng He, Si-ji Song, Tao Jiang, Zhan-song Zhou

**Affiliations:** 1 Department of Urology, Urologic Institute of PLA, Southwestern Hospital, Third Military Medical University, Chongqing, China; 2 Department of Urology, Kunming General Hospital of Chengdu Military Command, Kunming, Yunan, China; University College London, United Kingdom

## Abstract

**Purpose:**

Transurethral resection of the prostate (TURP) has a high failure rate in patients with small volume benign prostate hyperplasia (BPH) with bladder outlet obstruction (BOO). We describe and report the results of an alternative surgical method, selective transurethral resection of the prostate (STURP) in combination with transurethral incision of the bladder neck (TUIBN).

**Methods:**

Patients were randomized to receive TURP or STRUP+TUIBN in combination with TUIBN. Maximum urinary flow rate (Qmax), voided volume, and post voiding residual volume (PVR) were assessed at baseline and at 1, 3, and 6 months after surgery. Efficacy of treatment was assessed by lower urinary tract symptoms and IPSS.

**Results:**

Sixty three patients received STRUP+TUIBN and 61 received TURP. Surgical time, amount of prostate tissue resected, and blood loss was the same in both groups (all, *p*>0.05). The mean duration of follow-up was 9.02 and 8.53 months in patients receiving TURP and STRUP+TUIBN, respectively. At 6 months postoperatively, IPSS was 4.26±1.22 and 4.18±1.47 in patients receiving TURP and STRUP+TUIBN, respectively (*p*>0.05), and the Qmax in patients receiving STRUP+TUIBN was markedly higher than in those receiving TURP (28.28±6.46 mL/s vs. 21.59±7.14 mL/s; *p*<0.05). Bladder neck contracture and urinary tract infections were observed in 3 and 5 patients receiving TURP, respectively, and none in STURP.

**Conclusions:**

STRUP+TUIBN may offer a more effective and safer alternative to TURP for small volume BPH patients.

## Introduction

Benign prostate hyperplasia (BPH) is a common disease in elderly males characterized by lower urinary tract symptoms such as frequency, urgency, and dysuria, and is present in approximately 40% of men 50 years of age and above. The socioeconomic impact of BPH can be better appreciated in light of the growing prevalence of the disease and the upward trend in life expectancy. China has a rapidly increasing aging population with approximately 20,000,000 men with BPH, and a significant proportion of these patients will require surgical treatment [1], [2].

Transurethral resection of the prostate (TURP) is the gold standard for surgical treatment of BPH. However, TURP for BPH patients has been hampered by a high failure rate to achieve the desired outcome of alleviating urinary tract symptoms and approximately 15% to 20% of patients may require a second surgery 10 years after TURP. Small volume BPH may cause bladder outlet obstruction (BOO), and TURP as a single therapy cannot adequately address the multiple causes of BOO caused by small volume BPH [3]. In addition, TURP is associated with a relatively long hospital stay of up to 5 days and thus increased medical costs. These issues have fueled interest in developing alternative surgical procedures that are more effective and safer for relieving obstruction and at the same time decrease morbidity, shorten hospitalization, and reduce medical cost.

Studies examining treatments specifically for small volume BPH are somewhat few in number, and those that have been performed have reported encouraging results for transurethral incision of the prostrate (TUIP) [4] and minimal transurethral prostatectomy plus bladder neck incision [5]. Dong et al. [6] compared TURP, TURP plus transurethral incision of the bladder neck (TUIBN), and TURP plus transurethral resection of the bladder neck (TURBN) for the treatment of small volume BPH and reported that TURP plus TURBN was more effective at alleviating symptoms than TURP plus TUIBN. Despite these findings, the surgical risks of TURP are present in each procedure and TURBN is more invasive than TUIBN. The recently developed techniques for treating BPH using laser that include greenlight photoselective vaporisation of the prostate (PVP) and holmium laser enucleation of the prostate (HoLEP) have shown promising results, though their specific efficacy for small volume BPH has yet to be determined [7], [8].

To improve the effectiveness of treating small volume BPH, and at the same time reduce the incidence of intra- and postoperative complications we designed a selective transurethral resection of the prostate (STURP) in combination with TUIBN. In this prospective, randomized, single center study we compared the efficacy of STRUP+TUIBN with TURP in relieving the symptoms of BOO in patients with small volume BPH.

## Subjects and Methods

### Subjects

Patients with small volume BPH who sought surgical treatment at our institution between July 2009 and June 2010 were recruited in this propsective, randomized, single center study. A subject was eligible for enrollment in the study if they met the following criteria: 1) At least 50 years of age and received a clinical diagnosis of BPH; 2) Capable of reading, understanding, and completing a symptom and Quality of Life (QoL) questionnaire; 3) Prostate gland volume 20 to 40 cm^3^ as determined by digital examination and transrectal ultrasonography; 4) An International Prostrate Symptom Score (IPSS) ≥20; 5) Failed conservative medical therapy and thus surgically indicated for TURP; 6) BOO on urodynamic study; 7) Normal urinary bladder function. Exclusion criteria were: 1) History or evidence of prostate cancer or bladder cancer; 2) Prostate-specific antigen (PSA) level >4.0 ng/mL; 3) Previous prostate surgery or other invasive procedures to treat BPH; 4) Diabetes mellitus, cerebrovascular events, and/or neurogenic diseases; 5) Was expected to move out of the area during the study period, rendering follow-up per protocol impractical; 6) Currently participating in a clinical trial or other research study.

### Surgical Procedure

After initial cystoscopy and examination with the patient under anesthesia, the patient was randomized to receive TURP or STURP in combination with TUIBN. TURP was performed in a standard manner. STURP was performed using an Olympus resectoscope. The prostate gland tissue was excised between 1 and 11 o’clock to reach the surgical capsule. The urethral membrane between 11 and 1 o’clock was preserved. Thereafter, the circular fibrous tissue was cut open at 3 and 7 o’clock to reach the adipose tissue and distally the colliculus seminalis using a needle-shaped electrode. At the end of the procedure, a 22F 3-way Foley catheter with a closed drainage system was inserted. All patients were treated postoperatively with continuous saline bladder irrigation until bleeding ended, and the catheter was typically removed at 72 h postoperatively. In all patients, a blood count and serum electrolytes were measured immediately after surgery. All patients received routine antibiotics and hemostasis.

### Clinical Evaluation

Physical examination including digital rectal examination of the prostate and laboratory investigations including PSA were performed at baseline and subsequent follow-up visits. Maximum urinary flow rate (Qmax), voided volume, and post voiding residual volume (PVR) were assessed at baseline and the follow-up visit. Prostate volume was measured by transrectal ultrasound as follows: anteroposterior (H) and transverse prostate diameters (W) were measured on largest transverse images, and the horizontal distance between the most proximal and distal prostate boundaries on midline sagittal scan was considered to be the longitudinal diameter (L). Prostate volumes were then estimated by assuming an ellipsoid shape using the following formula: prostate volume = π/6×H×W×L. Efficacy of treatment as reflected by lower urinary tract symptoms and symptom-specific QoL was evaluated using the IPSS (score range 0 to 35; mild 0–7; moderate 8–19; severe >20) and the QoL index score, with higher scores representing greater severity of QoL impairment, at baseline and the subsequent follow-up visits.

### Study Endpoints

Patients were followed up at 1, 3, 6, and 12 months after surgery and IPSS, QoL score, and urinary flow rate were determined at each visit. Primary endpoints of the study were IPSS and QoL score. Secondary outcomes included PSA level, PVR, Qmax, and major urinary events. These parameters were measured at baseline and 6 months postoperatively. Major urinary events included acute urinary retention, the need for a prostate biopsy, gross hematuria, acute urinary tract infection, urinary tract stricture, and prostate cancer. In addition, operation time, intraoperative blood loss, and hospital stay length, changes in hemoglobin and serum sodium, catheterization time, and all perioperative complications were recorded. TURP syndrome was defined as a sodium concentration of ≤25 mmol/L after TURP with 2 or more of the following symptoms or signs; nausea, vomiting, bradycardia, hypotension, hypertension, chest pain, mental confusion, anxiety, paresthesia, and visual disturbance.

### Ethics Statement

The study protocol was approved by the ethics review board of Urologic Institute of PLA, Southwestern Hospital, Third Military Medical University. We have obtained written informed consent from all study participants. All of the procedures were done in accordance with the Declaration of Helsinki and relevant policies in China.

### Statistical Analysis

Data were expressed as mean ± standard deviation and analyzed using the SPSS software version 15.0 (SPSS, Inc., Chicago, IL). All patients with a baseline assessment and at least 1 post-baseline assessment were included in the analyses. The primary outcome variables (IPSS and QoL) was analyzed by end-point analysis of mean changes from the baseline. Results were analyzed using the χ^2^ test, and a value of *p*<0.05 was considered statistically significant.

## Results

### Patient Demographic and Baseline Characteristics

A total of 124 patients with small volume BPH met the entry criteria and were included in the study. Their mean age was 67.42±6.31 years (range, 55 to 76 years), and the mean duration of BPH symptoms was 26.04±5.281.46±1.12 years. Sixty-three patients were randomized to receive STURP+TUIBN, and 61 patients were randomized to receive TURP. The demographic and disease characteristics of the patients are shown in Table 1. The mean PSA level was 1.46±1.12 ng/mL and the mean IPSS was 25.14±8.18. The mean prostate volume by transrectal ultrasound was 30.45±6.11 mL, mean Qmax was 7.22±3.17 mL/s, and the mean urinary flow rate was 4.41±2.68 mL/s with a PVR of 34.87±25.69 mL. The mean baseline QoL score was 4.89±0.79. There was no significant difference in any of the demographic and baseline disease characteristics in patients receiving TURP and those receiving STURP+TUIBN.

**Table 1:** Patient demographic and baseline disease characteristics.

	All patients	TURP	STURP+TUIBN
Number of patients	124	61	63
Age (y)	67.42±6.31	68.66±7.52	66.83±4.91^a^
Course of illness (mo)	26.04±5.28	26.59±7.13	25.77±5.38^a^
Prostate volume (mL)	30.45±6.11	29.91±4.96	31.54±6.93^a^
IPSS	25.14±8.18	25.56±7.65	24.73±8.32^a^
Qmax (mL/s)	7.22±3.17	7.15±3.38	7.24±2.15^a^
PVR	34.87±25.69	33.69±29.68	35.62±19.73
PSA	1.46±1.12	1.47±1.31	1.45±1.35

Data presented as mean±standard deviation or number.

IPSS, International Prostate Symptom Score; PSA, prostate-specific antigen; PVR, post voiding residual volume; Qmax, maximal urinary flow rate; STURP, selective transurethral resection of the prostate; TUIBN, transurethral incision of the bladder neck; TURP, transurethral resection of the prostate.

a *p*<0.05 vs. the TURP group.

### Surgical Outcomes

All procedures were performed without complications in both groups. The operation time was 18.78±8.17 min in patients receiving TURP and 21.32±5.33 min in patients receiving STURP+TUIBN, and there was no difference in operation time between the 2 groups (*p*>0.05) (Table 2). In addition, similar amounts of prostate tissues were resected in the 2 groups (TURP, 13.09±4.93 g vs. STURP+TUIBN, 12.41±4.25 g; *p*>0.05). The volume of blood loss was also similar in the 2 groups (TURP, 3.98±1.38 mL vs. STURP+TUIBN, 4.34±1.64 mL; *p*>0.05). The postoperative IPSS was 8.39±2.91 in patients receiving TURP and 7.25±3.18 in patients receiving STURP+TUIBN, and there was no difference between the 2 groups (*p*>0.05). However, the postoperative I-PSS in both groups was significantly lower than then preoperative IPSS (*p*<0.01). The Qmax in patients receiving STURP+TUIBN was markedly higher than that in patients receiving TURP (TURP, 18.46±5.79 mL/s vs. STURP+TUIBN, 24.33±7.64 mL/s; *p*<0.05). The length of hospital stay was 4.14±1.62 d in patients receiving TURP and 4.32±1.47 d in patients receiving STURP+TUIBN. Hemoglobin and electrolyte levels of the 2 groups are shown in Table 3.

**Table 2:** Surgical outcomes of patients with small volume BPH undergoing TURP or STURP+TUIBN.

	TURP	STURP+TUIBN
Operation time (min)	18.78±8.17	21.32±5.33
Amount of resected prostate tissue (g)	13.09±4.93	12.41±4.25
Bleeding volume (mL)	3.98±1.38	4.34±1.64
IPSS	8.38±2.91^a^	7.25±3.18
Qmax (mL/s)	18.46±5.79^a^	24.33±7.64a,b
PVR	10.15±6.11	9.46±6.29
Hospital stay (d)	4.14±1.62	4.32±1.47

Data presented as mean±standard deviation.

BPH, benign prostate hyperplasia; IPSS, International Prostate Symptom Score; PVR, post voiding residual volume; Qmax, maximal urinary flow rate; STURP, selective transurethral resection of the prostate; TUIBN, transurethral incision of the bladder neck; TURP, transurethral resection of the prostate.

a *p*<0.01 vs. preoperative Qmax.

b *p*<0.05 vs. the TURP group.

**Table 3:** Hemoglobin and electrolyte levels of patients with small volume BPH undergoing TURP or STURP+TUIBN.

	TURP		STURP+TUIBN	
	Preoperative	Immediately postoperative	Preoperative	Immediately postoperative
Hemoglobin (mg/dL)	127.95±17.22	126.59±12.65	125.81±27.66	126.15±13.26
Sodium (mmol/L)	139.91±12.88	142.39±17.63	141.62±7.82	139.38±4.17
Potassium (mEq/L)	3.98±0.32	4.11±0.19	4.05±0.27	4.07±0.21
Chloride (mEq/L)	104.22±7.89	102.27±4.61	98.65±7.28	101.49±8.25

Data presented as mean±standard deviation.

STURP, selective transurethral resection of the prostate; TUIBN, transurethral incision of the bladder neck; TURP, transurethral resection of the prostate.

### Follow-up

The mean duration of followed-up for patients who received TURP was 9.02±0.79 months and was 8.53±1.86 months for patients who received STURP+TUIBN. At 6 months postoperatively, the IPSS was 4.26±1.22 in patients receiving TURP and 4.18±1.47 in patients receiving STURP+TUIBN, and there was no difference between the 2 groups (*p*>0.05) (Table 4). However, the postoperative IPSS in both groups at 6 months was significantly lower than the preoperative and immediately postoperative values (*p*<0.01). The postoperative Qmax in both groups was significantly lower than the Qmax immediately following surgery (*p*<0.01). In addition, the IPSS in patients receiving STURP+TUIBN was markedly higher than that in patients receiving TURP (TURP, 21.59±7.14 mL/s vs. STURP+TUIBN, 28.28±6.46 mL/s; *p*<0.05). The QoL score was 1.56±0.86 and 1.49±0.71 in the TURP and STURP+TUIBN groups, respectively, at 6 months postoperatively, and the values were significantly lower than the baseline values.

**Table 4:** Performance and adverse events in patients with small volume BPH undergoing TURP or STURP+TUIBN.

	TURP	STURP+TUIBN
IPSS	4.26±1.22	4.18±1.47
Qmax (mL/s)	21.59±7.14^a^	28.28±6.46a,b
Adverse events		
Urinary tract infection	5	1
Urinary tract stricture	0	1
Bladder contracture	3	0

Data presented as mean±standard deviation or number.

BPH, benign prostate hyperplasia; IPSS, International Prostate Symptom Score; Qmax, maximal urinary flow rate; STURP, selective transurethral resection of the prostate; TUIBN, transurethral incision of the bladder neck; TURP, transurethral resection of the prostate.

a *p*<0.01 vs. preoperative Qmax.

b *p*<0.05 vs. the TURP group.

Bladder neck contracture and urinary tract infections were observed in 3 and 5 patients receiving TURP, respectively. Urinary tract infection and urinary tract stricture were noted each in 1 patient receiving STURP+TUIBN. No other adverse events were observed.

## Discussion

In this study we designed a procedure specifically for the treatment of small volume BPH, STRUP+TUIBN, to improve the effectiveness of treating small volume BPH, and at the same time reduce the incidence of intra- and postoperative complications. The results showed that the IPSS in patients receiving STRUP+TUIBN was markedly higher than that in patients receiving TURP while STRUP+TUIBN was comparable to TURP in operation time, volume of blood loss, and resected prostate gland mass.

BPH is prevalent among elderly men, and is found in 40% of men aged 60 years and above and 80% of men aged 80 years and above. The enlarged prostate compresses against the urinary tract and contributes to urinary tract infections and increases the likelihood of urolithiasis. Long term urinary tract obstruction causes hydronephrosis, which can ultimately lead to renal failure and even death. Surgical therapy is recommended for those who have failed conservative therapy. Currently, TURP is the primary surgical treatment for BPH [1], [2]. For some BPH patients, however, the outcome of TURP is not sufficient, especially those with small volume BPH. While studies have examined the use of conventional BPH treatments for patients with small volume BPH, there have been no treatments developed that are specifically designed for small volume BPH.

Prostate enlargement causes mechanical and dynamic obstruction in both small volume and large volume BPH. In a urodynamic study of 63 BPH patients, Yang et al. [9] found that prostate mass correlates with BOO in patients with a prostate mass ≥30 g; however, this correlation was not seen in patients with a prostate mass <30 g, suggesting that apart from prostate enlargement, other factors also contribute to BOO. These factors may play insignificant roles in large volume BPH, but marked roles in small volume BPH. Bladder neck contracture due to fibrosis, increased tension from circular fibers in the bladder neck, and chronic prostatitis are common physiopathological causes of small volume BPH [10]. Medical therapy with α1-adrenergic receptor blockers and 5-α reductase inhibitors. cannot address all the root causes of small volume BPH, and fails to deliver desirable outcomes in some of these patients for whom surgical treatment for BOO remains a viable option.

While TURP is the most commonly used surgical treatment for BPH, the compression by the enlarged prostate in small volume BPH does not play a predominant role in BOO. Even if adequate resection of prostate tissue is accomplished, improvement in BOO is limited. In addition, simple TURP does not address the issues of bladder neck contracture due to fibrosis, increased tension from circular fibers in the bladder neck, and chronic prostatitis, which are common physiopathological causes of small volume BPH [11], [12]. TURP may also inadvertently aggravate postoperative bladder neck contracture due to the thermal effect on bladder neck tissue by intraoperative hemostasis and the resection of bladder neck tissue [13]. In the current study, bladder neck contracture was seen in 3 patients (4.92%, 3/61) undergoing simple TURP, which is consistent with the literature [14].

To avoid the above issues associated with TURP for small volume BPH, we designed STURP, which have several advantages over TURP. First, STURP selectively preserves partial epithelia in the anterior wall of the urinary tract, which increases epithelialization of the urinary tract after surgical trauma, thus minimizing irritation of the surgical wound by urine and reducing scar formation. Second, apart from resection of prostate tissue, STURP effectively relieves bladder neck contracture and lowers bladder neck tension, thereby alleviating BOO, and also avoids the possibility of incisional adhesions from excessive residual gland tissue in transurethral incision of the prostate (TUIP). Third, STURP preserves the original fibrous tissue between the 2 incisions in the bladder neck and avoids fibrosis of the preserved tissue, further reducing the possibility of bladder neck contracture due to excessive scar formation.

In our follow-up of small volume BPH patients, we found that the IPSS in patients receiving STURP was markedly higher than that in patients receiving TURP while STURP was comparable to TURP in operation time, volume of blood loss, and resected prostate gland mass, suggesting that STURP may offer a more effective treatment for small volume BPH without an increase in operative parameters. Given the limited number of patients and length of follow up in this study, the efficacy of STURP for small volume BPH needs to be confirmed by future prospective controlled studies involving a greater number of small volume BPH patients with longer follow-up. STURP is simple to learn and easy to perform, and we believe that STURP may offer a more effective and safer alternative to TURP for small volume BPH patients.
